# Adding smartphone-based cognitive-behavior therapy to pharmacotherapy for major depression (FLATT project): study protocol for a randomized controlled trial

**DOI:** 10.1186/s13063-015-0805-z

**Published:** 2015-07-07

**Authors:** Norio Watanabe, Masaru Horikoshi, Mitsuhiko Yamada, Shinji Shimodera, Tatsuo Akechi, Kazuhira Miki, Masatoshi Inagaki, Naohiro Yonemoto, Hissei Imai, Aran Tajika, Yusuke Ogawa, Nozomi Takeshima, Yu Hayasaka, Toshi A. Furukawa, On behalf of steering committee of the Fun to Learn to Act and Think through Technology (FLATT) project

**Affiliations:** Department of Clinical Epidemiology, Translational Medical Center, National Center of Neurology and Psychiatry, 4-1-1 Ogawa-Higashi, Kodaira, Tokyo 187-8551 Japan; National Center for Cognitive Behavior Therapy and Research, National Center of Neurology and Psychiatry, 4-1-1 Ogawa-Higashi, Kodaira, Tokyo 187-8551 Japan; Department of Neuropsychopharmacology, National Institute of Mental Health, National Center of Neurology and Psychiatry, 4-1-1 Ogawa-Higashi, Kodaira, Tokyo 187-8551 Japan; Department of Neuropsychiatry, Kochi Medical School, Kochi University, Kohasu, Okoh-cho, Nankokushi, Kochi 783-8505 Japan; Department of Psychiatry and Cognitive-Behavioral Medicine, Nagoya City University Graduate School of Medical Sciences, Mizuho-cho, Mizuho-ku, Nagoya, 467-8601 Japan; Miki Clinic, 1-1-3 Hiranuma, Nishi-ku, Yokohama, 220-0023 Japan; Department of Neuropsychiatry, Okayama University Hospital, 2-5-1 Shikata-cho Kita-ku, Okayama, 700-8558 Japan; Department of Field Medicine, Kyoto University Graduate School of Medicine, 46 Shimoadachi-cho, Yoshida Sakyo-ku, Kyoto, 606-8501 Japan; Departments of Health Promotion and Human Behavior, Kyoto University Graduate School of Medicine/School of Public Health, Yoshida Konoe-cho, Sakyo-ku, Kyoto, 606-8501 Japan

**Keywords:** Behavior therapy, Cognitive therapy, Computer-assisted therapy, Depression, Randomized controlled trials

## Abstract

**Background:**

Major depression is one of the most debilitating diseases in terms of quality of life. Less than half of patients suffering from depression can achieve remission after adequate antidepressant treatment. Another promising treatment option is cognitive-behavior therapy (CBT). However, the need for experienced therapists and substantive dedicated time prevent CBT from being widely disseminated.

In the present study, we aim to examine the effectiveness of switching antidepressants and starting a smartphone-based CBT program at the same time, in comparison to switching antidepressants only, among patients still suffering from depression after adequate antidepressant treatment.

**Methods/design:**

A multi-center randomized trial is currently being conducted since September 2014. The smartphone-based CBT program, named the “Kokoro-App,” for major depression has been developed and its feasibility has been confirmed in a previous open study. The program consists of an introduction, 6 sessions and an epilogue, and is expected to be completed within 9 weeks by patients. In the present trial, 164 patients with DSM-5 major depressive disorder and still suffering from depressive symptoms after adequate antidepressant treatment for more than 4 weeks will be allocated to the Kokoro-App plus switching antidepressant group or the switching antidepressant alone group. The participants allocated to the latter group will receive full components of the Kokoro-App after 9 weeks.

The primary outcome is the change in the total score on the Patient Health Questionnaire through the 9 weeks of the program, as assessed at week 0, 1, 5 and 9 via telephone by blinded raters. The secondary outcomes include the change in the total score of the Beck Depression Inventory-II, change in side effects as assessed by the Frequency, Intensity and Burden of Side Effects Rating, and treatment satisfaction.

**Discussion:**

An effective and reachable intervention may not only lead to healthier mental status among depressed patients, but also to reduced social burden from this illness. This paper outlines the background and methods of a trial that evaluates the possible additive value of a smartphone-based CBT program for treatment-resistant depression.

**Trial registration:**

UMIN-CTR: UMIN000013693 (registered on 1 June 2014)

## Background

Major depression is the second leading cause of deterioration of quality of life in humankind [1]. The economic loss due to depression is estimated at approximately 2 trillion yen per year in Japan [2]. Approximately 30,000 people a year die due to suicide in Japan. Half of these suicide victims are estimated to suffer from depression immediately before committing suicide [3].

The first-line treatment for depression in clinical settings is pharmacotherapy, especially antidepressant treatment; however, less than 50 % of patients receiving acute-phase antidepressant treatment for 2 to 4 months can achieve remission [4]. Other effective treatment options for depression include cognitive-behavior therapy (CBT); this has been shown to be as efficacious as pharmacotherapy [5] and to be more efficacious when combined with pharmacotherapy than pharmacotherapy alone [6]. CBT can, therefore, be a viable treatment option not only for patients preferring CBT to pharmacotherapy but also for patients still suffering from depression after an adequate trial of antidepressant treatment.

However, patients willing to receive CBT can rarely do so even in developed countries, because a typical course of CBT consists of sixteen 1-hour face-to-face sessions led by an experienced therapist. On the other hand, CBT delivered via the Internet or computers has been recently provided in Western countries, including Australia, the United Kingdom, the Netherlands and Sweden. The efficacy of computer-based or Internet-based CBT has been examined in previous systematic reviews. A systematic review and meta-analysis, combining results from 6 randomized controlled trials (RCTs) with 645 participants, estimated an effect size of computer-based CBT to be 0.78 (95 % confidence interval (CI): 0.59 to 0.63) in comparison with treatment as usual (TAU) or waiting-list controls [7]. Another systematic review combining results from 16 RCTs with 2807 participants showed that computer-based CBT led to a greater proportion of dropouts but to better efficacy at an effect size of 0.48 (0.33 to 0.63) in the short-term follow up than those in control conditions, whilst to neither superiority nor inferiority in the long term [8].

In the wake of recent developments in information and communication technology (ICT), CBT delivered via smartphones can be a better treatment option for depression than a computer-based one in terms of accessibility and portability. A clinical trial involving 52 participants in the community has shown that smartphone-based CBT was not inferior to computer-based CBT at 3-month follow up [9].

Given the vast number of patients with depression and the still very limited accessibility of effective CBT for them, it will be very meaningful and helpful to develop a CBT program taking advantage of this rapidly evolving ICT. In the present study, we aim to examine the effectiveness of adding a smartphone-based CBT program to switching antidepressants in comparison to that of switching antidepressants alone, for patients still suffering from depression after adequate antidepressant treatment. Among antidepressants, a systematic review and multiple-treatment meta-analysis evaluating the comparative efficacy of 12 newer antidepressants has suggested that escitalopram and sertraline are the most favorable in terms of efficacy and acceptability [10]. We aim to examine the effectiveness of switching from the previous antidepressant to escitalopram or sertraline and starting a smartphone-based CBT program at the same time, in comparison to switching to escitalopram or sertraline only, among patients still suffering from depression after adequate antidepressant treatment. We hypothesized that adding a smartphone-based CBT program to switching antidepressants could lead to greater improvement in depression symptoms among patients with treatment-refractory depression than switching antidepressants alone.

## Methods/design

### Trial design

A multi-center, parallel-arm, rater-blinded RCT has been planned. Participants who still suffer from full or residual major depressive disorder after adequate antidepressant treatment will be randomly allocated to either of the two intervention arms: 1) switching antidepressants to escitalopram or sertraline plus smartphone-based CBT program the “Kokoro-App” (“kokoro” means “mind” or “heart” in Japanese) consisting of 8 sections, or 2) switching antidepressants to escitalopram or sertraline only for 8 weeks after 1 week of introductory lead-in. The primary outcome is the slope of the Patient Health Questionnaire-9 (PHQ-9) through 0 to 9 weeks. The secondary outcomes include the slope of the Beck Depression Inventory (BDI-II) through 0 to 9 weeks; treatment satisfaction at 9 weeks; continuation of antidepressant pharmacotherapy up to 9 weeks; and the slope of the Frequency, Intensity and Burden of Side Effects Rating (FIBSER) through 0 to 9 weeks.

### Interventions

#### Smartphone-based CBT program: “Kokoro-App”

The smartphone-based CBT program the “Kokoro-App” for iPhones and iPads (Apple Inc., Cupertino, CA, USA) has been developed, based on an empirically supported CBT manual [11–13], and pilot tested for feasibility and acceptability. It consists of eight sections, including one introductory section, two sessions on self-monitoring, two sessions on behavioral activation, two sessions on cognitive restructuring, and one epilogue.

**Fig. 1:**
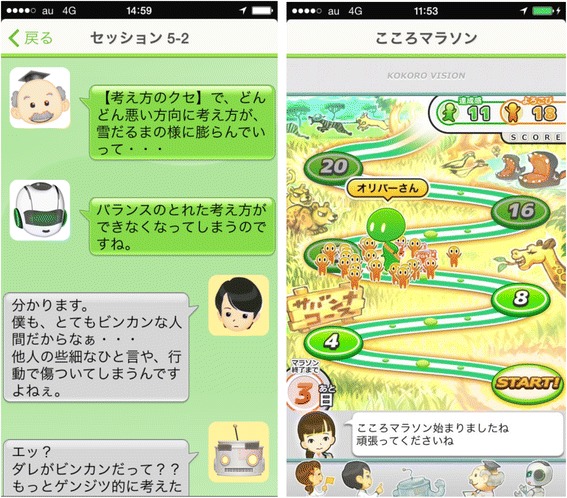
Screen shots from the Kokoro-App on an iPhone

The Kokoro-App can be completed within 7 weeks at the fastest. The main program consists of dialogues between characters who explain the principles and skills of CBT (Fig. 1). Homework needs to be completed by the participant between sessions. The participant can proceed to the next session 1 week after they start the previous one and after they finish the homework. One session needs approximately 30 minutes to complete. The central trial office sends an Email to each participant to encourage him/her to complete the session and the homework once every week during the program.

The participants and their attending physicians can check the entries made by the participants into the program by looking at the website (Kokoro-App Web). They can thus discuss the contents uploaded to the Kokoro-App Web during their consultations.

Security of the data exchanged through the Internet has been certificated by Secure Sockets Layer (SSL). An identification number and a password are required to enter the program or website when: 1) a participant logs in to the program at week 0; 2) a participant logs in to the Kokoro-App Web; and 3) the attending physician logs in to the Kokoro-App Web.

#### Antidepressant pharmacotherapy

In the present study, antidepressants that the participants had been taking before entry to the study will be switched to escitalopram or sertraline. A systematic review and a multiple-treatment meta-analysis have suggested that escitalopram and sertraline are the most favorable in terms of the efficacy and acceptability among 12 newer antidepressants [10]. The attending physician will start escitalopram or sertraline at entry (week 0) and aim to stop antidepressants other than these two by week 5 and to prescribe either 5–10 mg/day of escitalopram or 25–100 mg/day of sertraline at week 5.

#### Concomitant interventions

From week 0 through week 9, mood stabilizers (e.g., lithium, valproic acid, carbamazepine and lamotrigine), antipsychotics, electroconvulsive therapy, repetitive transcranial magnetic stimulation, individual CBT, and individual interpersonal therapy will not be allowed. Two antidepressant drugs may be prescribed until week 5 while the drug previously prescribed before entry is tapered and discontinued. After week 5, either escitalopram or sertraline should be prescribed as antidepressant monotherapy.

Any psychotherapy that is not specifically designed for depression, anxiolytics and hypnotics can be prescribed, and dosage of these can be changed between week 0 and week 9. Group psychotherapy during this period is allowed, but may not be changed or started.

The period after the assessment at week 9 is a follow-up phase, and any treatment is allowed at the attending physician’s discretion as TAU.

### Participants

The inclusion criteria for the participants are:

1. Men or women aged between 25 and 59 years upon entry into the study. We will limit participants to those aged 25 or older because patients younger than this age have been reported to be susceptible to increased suicidality after taking antidepressants in comparison with those taking placebo [14];
2. Primary diagnosis as major depressive disorder without psychotic features, according to the *Diagnostic and Statistical Manual of Mental Disorders, version 5* (DSM-5). This is confirmed by the treating psychiatrist through the semi-structured interview using the Primary Care Evaluation of Mental Disorders (PRIME-MD) [15]. It is not necessary that the patient satisfies the full criteria for a current major depressive episode upon entry. As long as the patient does not experience remission lasting for 2 months or more, the patient is considered to be in the same major depressive episode. Comorbid secondary diagnosis of anxiety disorders is allowed;
3. Not having taken either escitalopram or sertraline for the current episode;
4. Being antidepressant-resistant, defined as scoring 10 or more on the BDI-II at entry after taking one or more kinds of antidepressants at an adequate dosage for 4 or more weeks (corresponding to Stage I, II or III according to the criteria by Thase and Rush [16]), and it is judged by the attending physician that the patients should be switched to escitalopram or sertraline;
5. Taking only one kind of antidepressant at entry, and not taking any antipsychotics or mood stabilizers. Concomitant use of anxiolytics or hypnotics is allowed;
6. Willing to do the Kokoro-App program, and being judged suitable for the program by the attending physician;
7. Being used to smartphones, which is confirmed by the following conditions: a) the patient uses an iPhone, iPad (Apple Inc., Cupertino, CA, USA), Android smartphone or an equivalent in daily life; b) has an Email address for daily use; and c) has a mobile phone number for daily use;
8. Being an outpatient at entry, and having no plan to be hospitalized more than 1 week for any reason within 4 months;
9. Having no plan to transfer to a different hospital within 4 months;
10. Being able to respond to assessments about symptoms and side effects via telephone; and
11. Being able to understand and sign a written informed consent.

The exclusion criteria include:

1. Having taken any of the following interventions for the current episode: a) monoamine oxidase inhibitors (corresponding to Stage IV according to the criteria of Thase and Rush [16]); b) electroconvulsive therapy or repetitive transcranial magnetic stimulation (corresponding to Stage V [16]); c) both escitalopram and sertraline; or d) face-to-face individual CBT or face-to-face interpersonal psychotherapy;
2. Any of the following comorbid illnesses: a) past history of schizophrenia, schizoaffective disorder, or bipolar and related disorders according to DSM-5; or b) current diagnosis of neurocognitive disorders, feeding and eating disorders, substance-related and addictive disorders or borderline personality disorder;
3. Imminent risk of suicide as judged by the treating physician;
4. Physical illnesses possibly interfering with pharmacotherapy by escitalopram or sertraline, including: a) a possible prolonged QT syndrome, as judged through an interview with the attending physician; b) prolonged heart-rate corrected QT interval (QTc) in the electrocardiogram within 1 month (male: QTc > 450 ms, female: QTc > 470 ms [17]); c) taking medication known to prolong the QT interval and being judged unsuitable to take escitalopram or sertraline by the attending physician; d) severe or extremely unstable cardiovascular disease, such as current or past severe bradycardia, congestive heart failure or hypokalemia; e) severe or extremely unstable hepatic, renal, respiratory, blood, or endocrine function, or central nervous system disease or head injury; f) terminal stage of physical illnesses; g) currently taking pimozide; or h) history of hypersensitivity or allergy to escitalopram or sertraline;
5. Being currently pregnant or breastfeeding;
6. Currently participating in another clinical intervention study;
7. Family members living with the researchers of the present study; or
8. Being unable to understand the written Japanese language.

### Trial sites

A participating trial site must fulfill the following eligibility criteria:

1. Have a department of psychiatry or of psychosomatic medicine;
2. The principal trial physician and all the participating trial physicians at the site have understood the study protocol; and
3. The site is within mobile phone range.

On the other hand, a trial site will be ineligible if it satisfies one or more of the following conditions:

1. The principal trial physician withdraws consent for participating in the study;
2. No participant is registered for 6 months; or
3. The steering committee of the present trial judges the site to be inappropriate to recruit participants.

Initial trial sites include Nagoya City University Hospital in Aichi, Waseda Clinic in Gifu, Kochi University Hospital and Atago Hospital in Kochi, Japan.

### Assessment measures

#### Screening tools

##### Primary Care Evaluation of Mental Disorders (PRIME-MD)

Major depressive disorder, according to DSM-5, will be diagnosed through semi-structured interview using PRIME-MD [18] by the attending physicians at baseline.

##### The Beck Depression Inventory-II (BDI-II)

The BDI-II is a 21-item self-report instrument to measure the severity of depression. Its first version was developed in 1961 [19] and a major revision was undertaken in 1996 to make the scale more congruent with the modern diagnostic criteria for major depression [20]. Good reliability and validity have been reported for the Japanese version [21].

The time frame for evaluation is set to the past 2 weeks including the day of assessment. The BDI-II will be used for a screening tool at week 0 and participants with a total score of 10 or more will be included in the present study.

##### Electrocardiogram

The QTc will be checked using an electrocardiogram within 1 month before week 0. The QTc will be deemed prolonged if > 450 ms for males and > 470 ms for females, respectively, and will be checked again at week 1.

#### Primary outcome measure

##### Patient Health Questionnaire-9 (PHQ-9)

The PHQ-9 consists of the 9 diagnostic criteria items of the DSM-4 [15]. Each item is rated between 0 = “Not at all” through 3 = “Nearly every day,” making the total score range between 0 and 27. Excellent test-retest reliability and internal consistency reliability have been reported [15, 22]. Good construct validity has been demonstrated through associations with various severity indices [23]. The sensitivity to change is as good as or better than extant scales [24].

#### Secondary outc*o*me measures

##### Patients’ satisfaction with treatment

The following 2 questions will be graded on a scale of 100 by patients: 1) “To what extent has the treatment met your needs for these two months? Please answer on a scale of 1 to 100, presuming 60 as a passing mark”; and 2) “To what extent have you been satisfied with the treatment for these 2 months? Please answer on a scale of 1 to 100, presuming 60 as a passing mark.”

##### Continuation of protocol treatment up to week 9

In order to evaluate whether the Kokoro-App may have interfered with pharmacotherapy as defined in the protocol, continuation rates of protocol pharmacotherapy at week 9 will be compared between the intervention and control groups. Discontinuation of pharmacotherapy is defined as not taking a prescribed antidepressant for more than 1 week due to any reason as judged by the attending physician.

##### Frequency, Intensity, and Burden of Side Effects Rating (FIBSER)

FIBSER was originally used in STAR*D as a global rating scale for side effects. The FIBSER consists of 3 domains evaluating the frequency, intensity and severity of side effects, each of which has a score from 0 to 6. The reliability and validity of the FIBSER have been confirmed [25].

##### K6

K6 is a very short (6-item) self-report questionnaire to screen for common mental disorders and to evaluate the severity of general psychological distress [26]. Good validity and reliability have been confirmed [27], and its area under the curve compared to “gold-standard” diagnoses of depressive and anxiety disorders was 0.94 [28]. The Japanese version has been validated [28].

### Procedures

#### Screening

**Fig. 2:**
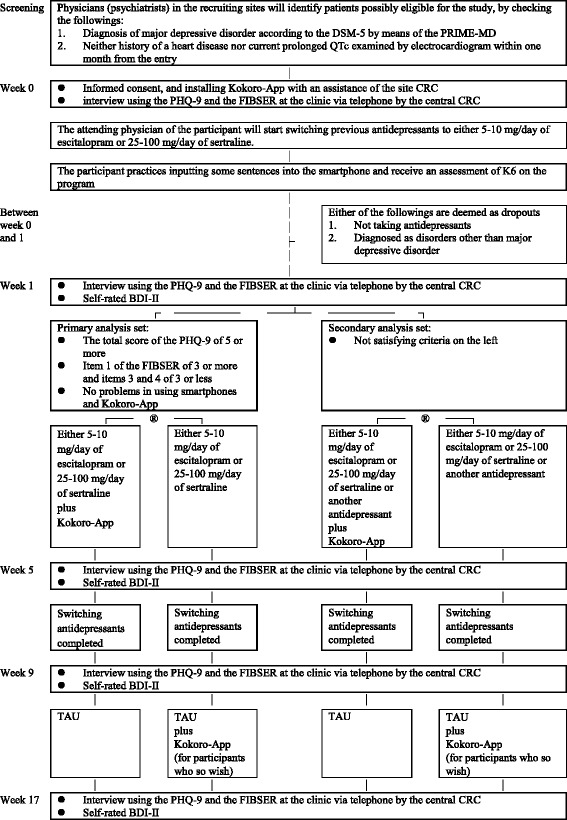
Flowchart of the trial. Abbreviations: BDI-II, Beck Depression Inventory-II; CRC, clinical research coordinator; FIBSER, Frequency, Intensity, and Burden of Side Effects Rating; PHQ-9, Patient Health Questionnaire-9; PRIME-MD, Primary Care Evaluation of Mental Disorders; TAU, treatment as usual

Physicians (psychiatrists) in the recruiting sites will identify patients possibly eligible for the study by checking diagnosis of major depressive disorder according to DSM-5 by means of the PRIME-MD. They will also confirm absence of a history of heart disease or current prolonged QTc by electrocardiogram within 1 month before entry, and check the other eligibility criteria (Fig. 2, Table 1).

**Table 1:** Schedule for the assessments

		Item	Week 0	Week 1	Week 5	Week 9	Week 17
		PRIME-MD	√				
		Basic characteristics^a^	√				
		Electrocardiogram	√	√			
		Informed consent	√				
		BDI-II	√	√	√	√	√
		PHQ-9	√	√	√	√	√
		FIBSER	√	√	√	√	√
		Blindness			√	√	√
		Treatment satisfaction				√	√
		Allocation		√			
Participant	K6	√	√	√		√	
			(Only those allocated to Kokoro-App)	(Only those allocated to Kokoro-App)		(Only those allocated to Kokoro-App)	

*BDI-II* Beck Depression Inventory-II, *CRC* clinical research coordinator, *FIBSER* Frequency, Intensity, and Burden of Side Effects Rating, *K6* 6-item self-report questionnaire to evaluate the severity of general psychological distress, *PHQ-9* Patient Health Questionnaire-9, *PRIME-MD* Primary Care Evaluation of Mental Disorders

^a^Basic characteristics include sex, age, education, work status, marital status, age at first depressive episode, number of episodes, duration of the current depressive episode, information about treatment for current episode, and current history of physical illnesses

#### Informed consent, installing the Kokoro-App and medication at week 0

A clinical research coordinator at a recruiting clinic (site CRC) will seek written informed consent from the candidate participant at the clinic at week 0. Participants will be informed that they can withdraw their consent at any time without stating the reason and that their withdrawal will not affect the medical services they receive. The written informed consent will be obtained along with the mobile phone number of the participant, with which the central trial office can contact him or her to assess the outcomes.

Immediately after consent, all participants will install the Kokoro-App onto his or her smartphone with an assistance of the site CRC. After successful installation, the server will provide a password specific to each participant via an Email to the participant. The participant will be able to proceed to the introductory section of the Kokoro-App by entering the password. In the introduction, the participant will practice entering some sentences into the smartphone and fill in a self-report measure of psychological distress (K6). All participants will receive an interview using the PHQ-9 and the FIBSER at the clinic via telephone from the central trial office.

The attending physician of the participant will start switching the previous antidepressant at week 0 to either 5–10 mg/day of escitalopram or 25–100 mg/day of sertraline. The patient will take escitalopram if he or she has taken sertraline for the current episode, and vice versa. If the patient has taken neither escitalopram nor sertraline for the current episode, one of these two will be selected by the attending physician. The maximum dosage of escitalopram was set at 10 mg/day because: 1) the efficacy of 20 mg/day of escitalopram has not been proved to be superior to that of 10 mg/day [29, 30]; 2) the tolerability of the former has been proved to be inferior to that of the latter in terms of dropout rates due to side effects [31]; and 3) a dose response has been reported between dosage of escitalopram and prolonged QT interval [32]. Anxiolytics and hypnotics can be used, started, increased/decreased and discontinued during the study.

#### Randomization and medication at week 1

An appointment at week 1 will be scheduled within 3 to 14 days from the entry. At week 1, the participant will receive an electrocardiogram and an interview, using the PHQ-9 and the FIBSER, via telephone from the central trial office. Based on these results, the participant will be classified into either of the following two groups: A) primary comparison set: those who have not responded or only partially responded to escitalopram or sertraline (defined by the total score of the PHQ-9 of 5 or more at week 1), are tolerant to escitalopram or sertraline (defined by item 1 of the FIBSER of 3 or more and items 3 and 4 of 3 or less at week 1, which means that the participant has been able to take the antidepressant for 3 days or more and the intensity and the interference with daily functions due to side effects of the medication is mild or less), and have no problems in using the smartphones and the Kokoro-App; B) secondary comparison set: those who have not satisfied any of the above criteria.

Within each group, the participants will be randomly allocated to either the combined smartphone-based CBT and antidepressant switch group (intervention group) or the antidepressant switch alone group (control group) at 1:1 ratio with a minimization method using the electronic data-capturing web program (EDC) at the central trial office. Random allocation will, therefore, be concealed. Clinics, number of antidepressants previously prescribed for the index episode (3 or more versus 2 or less), and a total score of the PHQ-9 (10 or more versus 9 or less) at week 1 will be used as stratification variables.

If the participant is allocated to the intervention group, he or she will be provided with a password to proceed to Session 1 of the Kokoro-App. If the participant is allocated to the control group, he or she will be informed that the Kokoro-App can be resumed if the participant is willing to do so after week 9.

With regard to antidepressants, if the participant is intolerant of escitalopram or sertraline prescribed at week 0 (defined by scores of 4 or more on either item 3 or 4 of FIBSER), the physician will suggest stopping the antidepressant and starting previous or new antidepressants other than escitalopram and sertraline.

#### Trial period: from week 1 through week 9

For all the participants, an appointment with the attending physician will be scheduled at least every 4 weeks. Visits must take place at weeks 5 and 9. The previous antidepressant must be tapered off by week 5, and either escitalopram or sertraline (or another antidepressant if the participant is intolerant of these two) must be prescribed as monotherapy at week 5. Only anxiolytics and hypnotics are allowed as psychotropic drugs other than antidepressants.

For the participants allocated to the intervention group, the physician can check the patient’s progress of the Kokoro-App through the Kokoro-App Web, and discuss it with the participant during consultations. If the participants allocated to the control group express interest in CBT by week 9, the physician is allowed to suggest self-help books about CBT but not to provide any specific CBT sessions.

At weeks 5 and 9, the participant will receive an interview, using the PHQ-9 and the FIBSER, via telephone at the clinic from the central trial office. Depression severity will also be assessed with the BDI-II. At week 9, an interview using a scale on treatment satisfaction will also be administered via telephone.

#### Follow-up period: from week 9 through week 17

After assessments at week 9, no restrictions will be posed in terms of medications, frequency of appointments, or CBT. The participants allocated to the control group can resume the Kokoro-App if they wish to. At week 17, all the participants will receive an assessment of the PHQ-9, the FIBSER and the scale on treatment satisfaction via telephone from the central trial office as well as to fill in a self-rating scale of the BDI-II.

#### Stopping rules for participants

##### Dropouts from the trial

The participants will be excluded from the intention-to-treat (ITT) cohort of the trial, if it was not possible to randomize them at week 1 or if their primary diagnosis was changed before week 1.

##### Deviation from protocol treatment

The following cases will be considered deviation from the trial protocol; however, the participant will not be considered to have dropped out of the trial at this stage and will receive the protocol assessments:

1. When prohibited concurrent treatments or prescription of the intervention antidepressant above the maximum dosage defined in the protocol (10 mg/day for escitalopram, and 100 mg/day for sertraline) took place between week 0 and week 9;
2. When changes in treatment that are allowed to be co-administered but not to be changed between week 0 and week 9 took place;
3. If the participant cannot take any pills of sertraline or escitalopram due to side effects between week 1 and week 9;
4. If the participant develops a manic/hypomanic/mixed episode, or is diagnosed with schizophrenia or dementia between week 1 and week 9.

##### Discontinuation of protocol treatment

If the participant meets any of the following conditions, the trial physician can stop the antidepressant or the Kokoro-App. The participant, however, will not be considered to have dropped out of the trial at this stage and will receive the protocol assessments:

1. The participant wishes to stop the protocol treatment;
2. The trial physician judges that it is difficult to continue the protocol treatment because of serious side effects;
3. The trial physician judges that the risk outweighs the benefit in continuing the protocol treatment even when no serious side effect is reported;
4. The participant becomes pregnant and the trial physician judges that the risk outweighs the benefit in continuing the protocol treatment;
5. The trial physician judges that it is inappropriate to continue the protocol treatment for some other reason.

##### Stopping assessment

If the participant withdraws consent for assessments, he/she will not be followed up.

#### Blindness and reliability of assessment from the central trial office

The primary outcome (PHQ-9) and information about side effects (FIBSER) at weeks 0, 5, 9, and 17 and treatment satisfaction judged by participants at weeks 9 and 17 will be collected via telephone by central CRCs, who are kept blind to groups to which the participant has been allocated.

At week 5, 9 and 17 assessments, the blindness of the central CRCs as to the participant’s treatment will be assessed by having the CRCs guess the allocated treatment by selecting one from the following: 1) I strongly believe that the patient is allocated to the combination group; 2) I guess that the patient is allocated to the combination group but am not confident; 3) I cannot tell; 4) I guess that the patient is allocated to the antidepressant alone group but am not confident; and 5) I strongly believe that the patient is allocated to the antidepressant alone group.

The inter-rater reliability of the central CRCs will be examined by comparing the assessors’ ratings of audio recordings of the PHQ-9 and FIBSER, recorded in our previous study [11], with the original ratings. In addition, five recordings in the present study will be assessed again by another rater blinded to the original rating in order to evaluate the inter-rater reliability in the present study.

### Reporting of adverse events and protection of participants

#### Definition of adverse events

An adverse event is defined as any unwanted or unintended sign (including laboratory exams), symptom or disease seen in participants of the trial. According to the “*Ethical Guidelines for Clinical Studies: Questions and Answers*,” which is published by the Japanese Ministry of Health, Labor and Welfare, severe adverse events are defined as events leading to any one the following: a) death; b) threatened death; c) admission or prolongation of admission for treatment; d) enduring and severe impairment and dysfunction; or e) congenital anomaly.

When a serious adverse event occurs, the trial physician must notify the principal investigator within 48 hours, regardless of the causal relationship with the trial intervention. The principal investigator shall report to the institutional review board in Kyoto University Graduate School of Medicine within 72 hours, and notify all the co-principal investigators. The principal investigator must also notify all the collaborators at all the recruiting sites. The collaborators will take the necessary measures according to the information from the principal investigator. If it concerns an unforeseen serious adverse event, the principal investigator shall report it to the Ministry of Health, Labor and Welfare.

#### Foreseeable adverse events

##### Escitalopram

Frequent side effects include: nausea (23.8 %), somnolence (23.5 %), headache (10.2 %), dry mouth (9.6 %), dizziness (8.7 %), fatigue (7.1 %), diarrhea (6.2 %), etc.

Serious side effects include: convulsion (unknown frequency), the syndrome of inappropriate anti-diuretic hormone secretion (SIADH) (unknown frequency), serotonin syndrome (unknown frequency), prolonged QT (unknown frequency), and ventricular tachycardia (unknown frequency).

##### Sertraline

Frequent side effects include: nausea (18.9 %), somnolence (15.2 %), dry mouth (9.3 %), headache (7.8 %), diarrhea (6.4 %), dizziness (5.0 %), etc.

Serious side effects include: serotonin syndrome (unknown frequency), malignant syndrome (unknown frequency), convulsion (unknown frequency), coma (unknown frequency), liver dysfunction (unknown frequency), SIADH (unknown frequency), Lyell syndrome and toxic epidermal necrolysis (unknown frequency), and anaphylactoid symptoms (unknown frequency).

##### Kokoro-App

No specific adverse events are presumed in participants who use the Kokoro-App. However, using the Kokoro-App might lead to psychological distress in some participants depending on their psychological state.

#### Compensation insurance

Because all the protocol antidepressant interventions are administered within the approved regulations in Japan, any health hazards shall be covered by the National Health Insurance. However, because the trial involves random allocation, we have contracted a private health insurance (Tokio Marine and Nichido Fire Insurance Co., Ltd.) to compensate for health hazards that have arisen due to this trial.

### Data monitoring

The trial will be supervised by the Data and Safety Monitoring Board (DSMB). The board consists of three independent experts in psychiatry and clinical trial methodology. The members of the committee are independent from the present study: Dr Teruhiko Higuchi (Chair of the DSMB, Psychiatrist, National Center for Neurology and Psychiatry), Professor Yoshio Hirayasu (Psychiatrist, Yokohama City University) and Dr Akiko Kada (Biostatistician, National Hospital Organization Nagoya Medical Center). The purpose of DSMB is to check the data monitoring reports prepared by the data center and make recommendations to the principal investigator, where necessary.

### Ethical issues

The present study is subject to the ethical guidelines for clinical studies published by the Japanese Ministry of Health, Labor and Welfare, as well as the ethical principles established for research on human beings as stipulated in the *Declaration of Helsinki* and further amendments thereto.

The protocol has been approved by the institutional review boards of Kyoto University Medical School on 12 June 2014 (ID: C842), of Nagoya City University on 11 August 2014 (ID: 45-14-0009) and of Kochi Medical School on 10 September 2014 (ID: ERB-100826). If important protocol modifications such as changes to eligibility criteria, outcomes, or analyses are needed, the investigators will discuss them and report to the review boards for approval.

Written informed consent will be obtained from all participants included in this study. Data of each participant will be handled with sequentially allocated numbers to maintain participant confidentiality.

### Data analysis

Details of the planned analyses for the trial will be given in the Statistical Analysis Plan, to be drafted by the trial statistician. The analyses will be conducted according to the plan.

#### Primary analyses

The primary outcome will be analyzed using a mixed model with repeated measures to examine treatment effect parameters of all the eligible subjects in the primary comparison set according the ITT principle. Allocation group (intervention) and stratification variables used in randomization will be incorporated into the model. A regression coefficient (beta), its 95 % CIs, and a 2-side *P* value will be calculated. The statistical significance is set at 0.05 (2-sided). If the changes are not linear, an appropriate model will be applied.

#### Secondary analyses

We will perform secondary analyses to supplement our primary analysis and to obtain finer understanding of our clinical questions. The secondary analyses will use models similar to those of the primary analysis, will analyze data from both the primary and secondary comparison sets as well as from the per protocol set, and will also examine data for the secondary outcome measures. These analyses will be conducted for exploratory purposes. We will not use adjustment for multiple tests. We will report the effect sizes and their 95 % CIs. The methods for these secondary analyses will be stated in detail in the Statistical Analysis Plan.

#### Interim analyses

We will not perform interim analyses.

### Sample size

Sample size was based on a power analysis with 0.8 power to detect an effect size of 0.5 between the groups at *P* = 0.05 (2-sided). It was calculated that 63 patients would be required for each of the two arms for the primary comparison set. Assuming that 30 % of the initial entries would drop out or otherwise be classified into the secondary comparison set at week 1, 164 participants would need to be recruited into the trial.

The effect size of the Kokoro-App was estimated at 0.5 because we anticipated a medium effect size [33] compared with the wait-list control group. In fact, an effect size of 0.69 was observed when we conducted a clinical trial of a 1-to-1 telephone-based CBT program, which formed the prototype of the Kokoro-App, in comparison with a wait-list control [11]. Systematic reviews of clinical trials on the efficacy of computer or Internet-based CBT showed 0.49 [8] and 0.78 [7] in the acute-phase treatment. We therefore calculate that we could anticipate an effect size of approximately 0.5 for the Kokoro-App.

The primary outcome of the present trial is the change in the PHQ-9 through weeks 0, 1, 5 and 9. Taking account of the sample size calculation for repeated measures [34], a sample size per group will not exceed 63 even when we consider associations of 4 time points. However, this is derived from presumption that the treatment effect is stable between any two time points. If there is a large difference between time points, statistical power would decrease. If this does occur, we will recalculate and examine the achieved statistical power post hoc.

### Funding

This study is funded by the Pragmatic Psychopharmacotherapy Research Project initiated by the Japan Foundation for Neuroscience and Mental Health. The Project has received donations from Asahi Kasei Pharma Corp., Eli Lilly Japan KK, GlaxoSmithKline KK, Ltd., Janssen Pharmaceuticals KK, Meiji Seika Pharma Co., Mitsubishi Tanabe Pharma Corporation, MSD KK, Mochida Pharmaceutical Co., Ltd., Otsuka Pharmaceutical Co., Ltd., Taisho Pharmaceutical Co., Ltd., Pfizer Japan Inc., Ltd. and Shionogi & Co. The up-to-date information will be uploaded on the trial website (http://ebmh.med.kyoto-u.ac.jp/flatt/) and will be provided in the informed consent document for the participant.

### Publication policy

The results from the study will be submitted to peer-reviewed journals. The collaborating researchers have the right to be named as the first author of these papers in the order of their number of recruitment. TAF will be the corresponding author for all the papers.

Trial principal physicians, trial participating physicians and other members of the Steering Committee, if they do not appear as co-author, will be listed at the end of the article. Such authors may be counted as co-authors in some journals but not in others.

### Study period

The study period of this trial will be between September 2014 and March 2017, with the participant entry period between June 2014 and October 2016.

## Discussion

To our knowledge, the present study represents the first trial investigating the efficacy of smartphone-based CBT in clinical settings, especially for patients still suffering from depression after an adequate trial of antidepressant treatment. Considering the high dropout rate of computer-based CBT [8], smartphone-based CBT may offer a more accessible option because patients can access it anywhere and anytime they have a chance and are willing to do so. Only fewer than 50 % of patients receiving acute-phase antidepressant treatment can achieve remission [4], so that easily accessible CBT may offer some additional benefits in the treatment of depression. The present study focuses on patients with antidepressant-resistant depression, so that targeted population may be those in most need. If the efficacy of a smartphone-based CBT program in this population is confirmed, applicability of the program in real clinical settings is quite promising.

The present study is, however, not without some methodological limitations. First of all, not all patients who are interested in and willing to do the Kokoro-App have a smartphone. This may undermine the applicability of the results from this trial to all patients with antidepressant-resistant depression. Especially, the results might not be applicable to patients in developing countries and to patients with poor ICT literacy.

Second, we selected a wait-list control as the comparator due to the feasibility and ethical considerations, but the nocebo effects of the latter condition can play an important role in the estimation of the efficacy of the smartphone-based CBT. In a network meta-analysis of CBTs, waiting-list controls and no treatment controls, the odds ratio of response for no treatment over waiting-list was statistically significant at 2.9 (95 % CI: 1.3 to 5.7) [35]. However, in the present trial we planned to switch a previous antidepressant treatment to a newer drug for each patient (e.g., sertraline or escitalopram) for all participants at entry to the study, thus raising expectation across the intervention and control arms. This might lead to decreasing nocebo effects of the waiting-list control.

Third, one may consider that results from the primary comparison set are those of an efficacy trial not of an effectiveness trial, because we would only include patients who are able to tolerate the trial antidepressant for 1 week and are accustomed to using a smartphone. This decision was made a priori because the trial is the first one of this kind. However, we will include patients who had failed to meet these conditions in our secondary comparison set, and results from these analyses can be utilized in future effectiveness trials.

Fourth, although the effect size of 0.5 used for calculation of sample sizes was estimated from results of the previous studies on the efficacy of computer or Internet-based CBT [7, 8], one may think that the effect size is too optimistic because the present study focuses on patients with treatment-refractory depression. In addition, a majority of the control conditions used in trials included in previous systematic reviews [7, 8] were waiting-list, which might have led to plausibly large effect sizes. However, our previous clinical trial comparing the efficacy of a telephone-based CBT program added to the employee assistance program with that of the latter alone showed an effect size of 0.69 [11]. The smartphone-based CBT program in the present study was based on this telephone-based program, and we considered that the estimated effect size of 0.5 in the present study was reasonable.

## Trial status

The randomized trial, which commenced in September 2014, is currently in the phase of participant enrollment and follow up.

## Abbreviations

BDI-II: Beck Depression Inventory-II
CI: Confidence interval
CRC: Clinical research coordinator
CBT: Cognitive-behavior therapy
DSM: Diagnostic and Statistical Manual of Mental Disorders
DSMB: Data and Safety Monitoring Board
EDC: Electronic data capturing
FIBSER: Frequency, Intensity, and Burden of Side Effects Rating
ICT: Information and communication technology
ITT: Intention-to-treat
PHQ-9: Patient Health Questionnaire-9
PRIME-MD: Primary Care Evaluation of Mental Disorders
QTc: Heart-rate corrected QT interval
RCT: Randomized controlled trial
SIADH: Syndrome of inappropriate anti-diuretic hormone secretion
SSL: Secure Sockets Layer
TAU: Treatment as usual

## References

[1] Murray CJ, Vos T, Lozano R, Naghavi M, Flaxman AD, Michaud C (2012). Disability-adjusted life years (DALYs) for 291 diseases and injuries in 21 regions, 1990–2010: a systematic analysis for the Global Burden of Disease Study 2010. Lancet.

[2] Sado M, Yamauchi K, Kawakami N, Ono Y, Furukawa TA, Tsuchiya M (2011). Cost of depression among adults in Japan in 2005. Psychiatry Clin Neurosci.

[3] Arsenault-Lapierre G, Kim C, Turecki G (2004). Psychiatric diagnoses in 3275 suicides: a meta-analysis. BMC Psychiatry.

[4] Trivedi MH, Rush AJ, Wisniewski SR, Nierenberg AA, Warden D, Ritz L (2006). Evaluation of outcomes with citalopram for depression using measurement-based care in STAR*D: implications for clinical practice. Am J Psychiatry.

[5] Cuijpers P, van Straten A, van Oppen P, Andersson G (2008). Are psychological and pharmacologic interventions equally effective in the treatment of adult depressive disorders? A meta-analysis of comparative studies. J Clin Psychiatry.

[6] Cuijpers P, Dekker J, Hollon SD, Andersson G (2009). Adding psychotherapy to pharmacotherapy in the treatment of depressive disorders in adults: a meta-analysis. J Clin Psychiatry.

[7] Andrews G, Cuijpers P, Craske MG, McEvoy P, Titov N (2010). Computer therapy for the anxiety and depressive disorders is effective, acceptable and practical health care: a meta-analysis. PLoS One.

[8] So M, Yamaguchi S, Hashimoto S, Sado M, Furukawa TA, McCrone P (2013). Is computerised CBT really helpful for adult depression? A meta-analytic re-evaluation of CCBT for adult depression in terms of clinical implementation and methodological validity. BMC Psychiatry.

[9] Watts S, Mackenzie A, Thomas C, Griskaitis A, Mewton L, Williams A (2013). CBT for depression: a pilot RCT comparing mobile phone vs. computer. BMC Psychiatry.

[10] Cipriani A, Furukawa TA, Salanti G, Geddes JR, Higgins JP, Churchill R (2009). Comparative efficacy and acceptability of 12 new-generation antidepressants: a multiple-treatments meta-analysis. Lancet.

[11] Furukawa TA, Horikoshi M, Kawakami N, Kadota M, Sasaki M, Sekiya Y (2012). Telephone cognitive-behavioral therapy for subthreshold depression and presenteeism in workplace: a randomized controlled trial. PLoS One.

[12] Ludman EJ, Simon GE, Tutty S, Von Korff M (2007). A randomized trial of telephone psychotherapy and pharmacotherapy for depression: continuation and durability of effects. J Consult Clin Psychol.

[13] Simon GE, Ludman EJ, Rutter CM (2009). Incremental benefit and cost of telephone care management and telephone psychotherapy for depression in primary care. Arch Gen Psychiatry.

[14] Stone M, Laughren T, Jones ML, Levenson M, Holland PC, Hughes A (2009). Risk of suicidality in clinical trials of antidepressants in adults: analysis of proprietary data submitted to US Food and Drug Administration. BMJ.

[15] Spitzer RL, Kroenke K, Williams JB (1999). Validation and utility of a self-report version of PRIME-MD: the PHQ primary care study. Primary care evaluation of mental disorders. Patient health questionnaire. JAMA.

[16] Thase ME, Rush AJ (1997). When at first you don’t succeed: sequential strategies for antidepressant nonresponders. J Clin Psychiatry.

[17] Vetter VL (2007). Clues or miscues? How to make the right interpretation and correctly diagnose long-QT syndrome. Circulation.

[18] Spitzer RL, Williams JW, Kroenke K, Linzer M, deGruy FV, Hahn SR (1994). Utility of a new procedure for diagnosing mental disorders in primary care: the prime-md 1000 study. JAMA.

[19] Beck AT, Ward CH, Mendelson M, Mock J, Erbaugh J (1961). An inventory for measuring depression. Arch Gen Psychiatry.

[20] Beck AT, Steer RA, Brown GK (1996). BDI-II: Beck Depression Inventory, Second Edition, Manual.

[21] Hiroe T, Kojima M, Yamamoto I, Nojima S, Kinoshita Y, Hashimoto N (2005). Gradations of clinical severity and sensitivity to change assessed with the Beck Depression Inventory-II in Japanese patients with depression. Psychiatry Res.

[22] Pinto-Meza A, Serrano-Blanco A, Penarrubia MT, Blanco E, Haro JM (2005). Assessing depression in primary care with the PHQ-9: can it be carried out over the telephone?. J Gen Intern Med.

[23] Kroenke K, Spitzer RL, Williams JB (2001). The PHQ-9: validity of a brief depression severity measure. J Gen Intern Med.

[24] Lowe B, Unutzer J, Callahan CM, Perkins AJ, Kroenke K (2004). Monitoring depression treatment outcomes with the Patient Health Questionnaire-9. Med Care.

[25] Wisniewski SR, Rush AJ, Balasubramani GK, Trivedi MH, Nierenberg AA (2006). Self-rated global measure of the frequency, intensity, and burden of side effects. J Psychiatr Pract.

[26] Kessler RC, Andrews G, Colpe LJ, Hiripi E, Mroczek DK, Normand SL (2002). Short screening scales to monitor population prevalences and trends in non-specific psychological distress. Psychol Med.

[27] Cornelius B, Groothoff J, van der Klink J, Brouwer S (2013). The performance of the K10, K6 and GHQ-12 to screen for present state DSM-IV disorders among disability claimants. BMC Public Health.

[28] Furukawa TA, Kawakami N, Saitoh M, Ono Y, Nakane Y, Nakamura Y (2008). The performance of the Japanese version of the K6 and K10 in the World Mental Health Survey Japan. Int J Methods Psychiatr Res.

[29] Hirayasu Y (2011). A dose-response and non-inferiority study evaluating the efficacy and safety of escitalopram in patients with major depressive disorder: a placebo- and paroxetine-controlled, double-blind, comparative study. Japanese J Clin Psychopharmacol.

[30] Hirayasu Y (2011). A dose-response study of escitalopram in patients with major depressive disorder: a placebo-controlled, double-blind study. Japanese J Clin Psychopharmacol.

[31] Cipriani A, Santilli C, Furukawa TA, Signoretti A, Nakagawa A, McGuire H (2009). Escitalopram versus other antidepressive agents for depression. Cochrane Database Syst Rev.

[32] Castro VM, Clements CC, Murphy SN, Gainer VS, Fava M, Weilburg JB (2013). QT interval and antidepressant use: a cross sectional study of electronic health records. BMJ.

[33] Cohen J (1988). Statistical power analysis in the behavioral sciences.

[34] Diggle PJ, Heagerty PJ, Liang K, Zeger SL (2002). Analysis of longitudinal data.

[35] Furukawa TA, Noma H, Caldwell DM, Honyashiki M, Shinohara K, Imai H (2014). Waiting list may be a nocebo condition in psychotherapy trials: a contribution from network meta-analysis. Acta Psychiatr Scand.

